# Guanylic nucleotide starvation affects *Saccharomyces cerevisiae *mother-daughter separation and may be a signal for entry into quiescence

**DOI:** 10.1186/1471-2121-6-24

**Published:** 2005-05-04

**Authors:** Isabelle Sagot, Jacques Schaeffer, Bertrand Daignan-Fornier

**Affiliations:** 1 Institut de Biochimie et Génétique Cellulaires, UMR CNRS 5095 – Université Victor Segalen / Bordeaux II 1, rue Camille Saint Saëns – F-33077 Bordeaux Cedex – France

## Abstract

**Background:**

Guanylic nucleotides are both macromolecules constituents and crucial regulators for a variety of cellular processes. Therefore, their intracellular concentration must be strictly controlled. Consistently both yeast and mammalian cells tightly correlate the transcription of genes encoding enzymes critical for guanylic nucleotides biosynthesis with the proliferation state of the cell population.

**Results:**

To gain insight into the molecular relationships connecting intracellular guanylic nucleotide levels and cellular proliferation, we have studied the consequences of guanylic nucleotide limitation on *Saccharomyces cerevisiae *cell cycle progression. We first utilized mycophenolic acid, an immunosuppressive drug that specifically inhibits inosine monophosphate dehydrogenase, the enzyme catalyzing the first committed step in *de novo *GMP biosynthesis. To approach this system physiologically, we next developed yeast mutants for which the intracellular guanylic nucleotide pools can be modulated through changes of growth conditions. In both the pharmacological and genetic approaches, we found that guanylic nucleotide limitation generated a mother-daughter separation defect, characterized by cells with two unseparated daughters. We then showed that this separation defect resulted from cell wall perturbations but not from impaired cytokinesis. Importantly, cells with similar separation defects were found in a wild type untreated yeast population entering quiescence upon nutrient limitation.

**Conclusion:**

Our results demonstrate that guanylic nucleotide limitation slows budding yeast cell cycle progression, with a severe pause in telophase. At the cellular level, guanylic nucleotide limitation causes the emergence of cells with two unseparated daughters. By fluorescence and electron microscopy, we demonstrate that this phenotype arises from defects in cell wall partition between mother and daughter cells. Because cells with two unseparated daughters are also observed in a wild type population entering quiescence, our results reinforce the hypothesis that guanylic nucleotide intracellular pools contribute to a signal regulating both cell proliferation and entry into quiescence.

## Background

Guanylic nucleotides are critical for multiple crucial cellular processes such as replication, transcription, translation and signalization *via *small GTPases. Most cell types recycle GMP from guanosine or guanine, which are either taken up from the surrounding environment or synthesized by the intracellular metabolism. GMP can also be synthesized de novo from IMP *via *two consecutive enzymatic steps, both highly conserved through evolution. Initially, the inosine monophosphate dehydrogenase (IMPDH) catalyzes conversion of IMP into XMP, the first committed step in *de novo *GMP biosynthesis. Subsequently, GMP synthetase converts the newly produced XMP into GMP.

The expression of IMPDH encoding genes is tightly regulated. In both yeast and mammalian cells, high guanylic nucleotide levels repress the transcription of IMPDH encoding genes [1,2]. More strikingly, the transcription of IMPDH encoding genes in mammals is linked to cellular proliferation. In non-dividing cells, the expression level of IMPDHs is low, whereas it is highly increased in actively proliferating cells such as cancerous cells [3-6]. Further, IMPDH over-expression bypasses the anti-proliferative effect of p53, indicating that this p53 function requires proper control of IMPDH activity [7].

*Saccharomyces cerevisiae *cells have two major sources of guanylic nucleotides: intracellular IMP and extracellular guanine. Therefore GMP synthesis fully relies on IMPDH and GMP synthetase activities in the absence of guanine in the growth medium. Consistently, mutations in these cognate genes lead to guanine auxotrophy [8,9]. Similar to mammalian cells, *S. cerevisiae *genes involved in GMP biosynthesis are highly expressed during exponential growth, but are actively repressed through specific regulatory sequences when cells arrest proliferation upon nutrient limitation [10]. Moreover, the intracellular GTP/GDP ratio drastically decreases when yeast cells enter a quiescent state [11]. These experiments lead to the appealing hypothesis that intracellular guanylic nucleotides levels contribute to a signal regulating cell proliferation. However, the molecular pathways linking cell cycle progression to IMPDH activity, and thus to intracellular guanylic nucleotide pools remain unknown.

Mycophenolic acid (MPA) is a well-characterized non-competitive and reversible inhibitor of IMPDH that severely depletes intracellular GTP pool down to 10% of normal level [12,13]. MPA reduces or even abrogates proliferation of various cell types [14,15]. MPA particularly affects lymphocyte division and because it has few secondary effects, its pro-drug form, the mycophenolate mofetil (Cellcept, Roche), is in path to replace cyclosporine A as a commonly used immunosuppressive drug. At the cellular level, MPA inhibits lymphocyte cell cycle progression by arresting cells in G1. Although this effect correlates with the depletion of guanylic nucleotide pools [13,16], addition of guanosine and 8-aminoguanosine, which can partially replenish guanylic nucleotide pools, does not allow MPA treated cells to re-enter G2/M [17]. Thus, MPA treatment, although apparently blocking cells in G1, could also affect later steps of the cell cycle. In budding yeast, MPA treatment slows cell proliferation and causes various effects on gene expression and thus on the yeast proteome [18]. In a previous study, we have shown that MPA affects yeast cell size, DNA content, budding pattern and causes occasional perturbations of actin and microtubule cytoskeletons [18]. In addition, several mutants affected for various cellular functions are hypersensitive to MPA [19]. However, these data do not point at an obvious molecular process that would account for the effects of MPA on cell proliferation.

Here, to gain insight into the molecular relationships between intracellular guanylic nucleotide levels and cell cycle progression, we studied the effects of MPA treatment on *Saccharomyces cerevisiae *cell cycle progression. We first demonstrated that, although cells did not arrest in a particular cell cycle stage (confirming that there was no checkpoint for guanylic nucleotides in yeast), a large proportion of the population was slowed in telophase. We further observed that many MPA treated cells presented two unseparated daughter cells. We have shown that this specific morphology was due to a defect in mother-daughter separation and that it was probably a consequence of cell wall perturbations. To validate the results obtained with MPA, we developed yeast mutants in which guanylic nucleotide pools could be modulated by the composition of the growth medium. Using these genetic tools, we confirmed that cell separation was indeed the cell cycle step mostly perturbed by guanylic nucleotides starvation. Finally, the observation that cells entering quiescence also displayed the characteristic "two-daughter cells" morphology strongly suggests that a decrease in intracellular guanylic nucleotide levels may be part of a signal for yeast cells to enter stationary phase.

## Results

### Mycophenolic acid treatment particularly affects the last step of the yeast cell cycle

**Figure 2:**
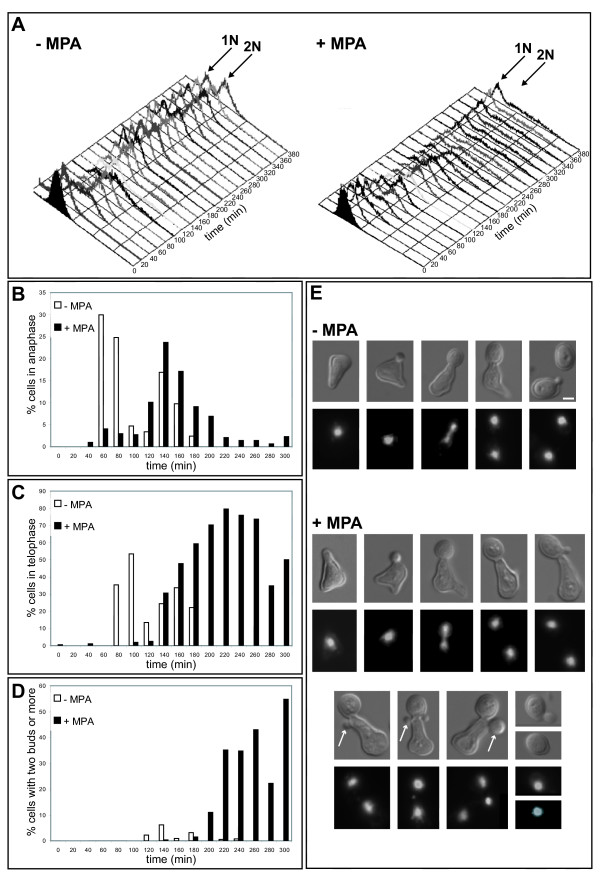
**Effects of MPA on the yeast cell cycle progression**. **A**. Cell cycle progression of a yeast cell population synchronized in G1 with alpha factor and released in the absence (left) or in the presence of 100 μg/mL MPA (right) analyzed by FACS. **B**. Percentage of cells in anaphase in function of the progression through the cell cycle. Cells are the same as in A. More than 200 cells were counted for each time point. Cells were scored as being in anaphase when the mother cell DNA mass was clearly still connected to the DNA mass of the daughter cell, typically as the third cell shown in the – MPA panel of figure 2E. **C**. Percentage of cells in telophase in function of the progression through the cell cycle. Cells are the same as in A. More than 200 cells were counted for each time point. Cells were scored as being in telophase when displaying two clearly separated DNA masses, typically as the fourth cell shown in the – MPA panel of figure 2E. **D**. Percentage of cells with two or more daughter cells in function of the progression through the cell cycle. Cells are the same as in A. More than 200 cells were counted for each time point. **E**. Cells representative of each cell cycle stage (phase contrast and propidium iodide staining of the nucleus) for the untreated (top panel) or MPA treated (bottom panel) population. The unusual elongated cell morphology is due to the alpha factor treatment. Arrows indicate examples of second daughter cell appearing while the first daughter cell is not yet separated from the mother cell. Bar: 2 μm.

**Figure 3:**
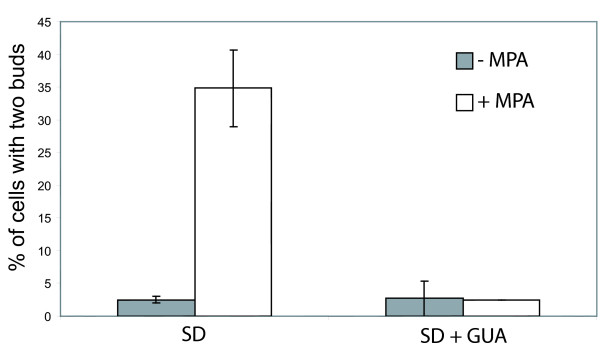
**Effects of MPA treatment on an unsynchronized yeast cell population**. Percentage of budding cells with two daughter cells after 4 hours growth in SD medium without (left) or with guanine (right) in the absence (grey bars) or in the presence (white bars) of 100 μg/mL MPA. More than 200 cells were counted for each condition.

MPA treatment affects yeast growth in a concentration-dependent manner [18]. This growth defect can result from either a general slowing down of the entire cell cycle or a pause in a specific cell cycle step. To gain insight into this issue, wild type yeast cells were synchronized in G1 using alpha factor and released in either the absence or the presence of 100 μg/mL of MPA. At such a concentration, MPA does not affect cell viability nor totally arrest cell growth [18]. Higher MPA concentration gave similar effects, most probably because yeast cells detoxified the drug [19]. We then monitored cell cycle progression by FACS. As shown in figure 2A, although progression through initial cell cycle steps was slower for MPA treated cells, the population lagged most predominantly in a 2N DNA content stage. Fluorescence microscopy revealed that although for MPA-treated cells the anaphase onset was slightly delayed, its duration was almost similar to the anaphase of control cells (Fig. 2B). In fact, treated cells were mostly pausing in a stage where the DNA masses were totally separated (telophase, Fig. 2C). To our surprise, in the treated population we observed mother cells with two apparent daughter cells (Fig. 2D and 2E). The cells with two apparent daughters represented more than 50% of the population 300 minutes after the release from G1. To confirm this result, a non-synchronized population was treated with MPA. After 4 hours in the presence of MPA, more than 30% of the budding cells displayed two apparent daughters (Fig. 3), a phenotype we refer to as "bibudded", for simplicity.

A "bibudded" phenotype could result either from a single mother cell with two daughter cells, or from an unrelated G1 cell "sticking" to a normally budding cell. Since MPA treated cells were found to be sensitive to sonication, we used a fluorescence-based approach to distinguish between these two possibilities. Equal amounts of cells over-expressing a green variant of GFP were mixed with cells over-expressing a blue variant of GFP, grown to OD_600 nm _0.2 and then treated with MPA.

After 4 hours of incubation, less than 1% (0.3% ± 0.3, N>100 for each of 4 independent experiments) of "bibudded" cells displayed one apparent daughter expressing a different GFP variant than its joined budding cell. Thus, more than 99% of the cells with two apparent daughters were monocolor, which is far more than the 50% expected for a random population of false "bibudded" cells. This experiment demonstrated that the population is indeed composed of a large proportion of cells with two attached daughters. In conclusion, MPA treatment particularly slowed down telophase and caused the appearance of cells with two unseparated daughters.

### MPA effects are reversed by extracellular guanine and are not a consequence of translation inhibition

**Figure 1:**
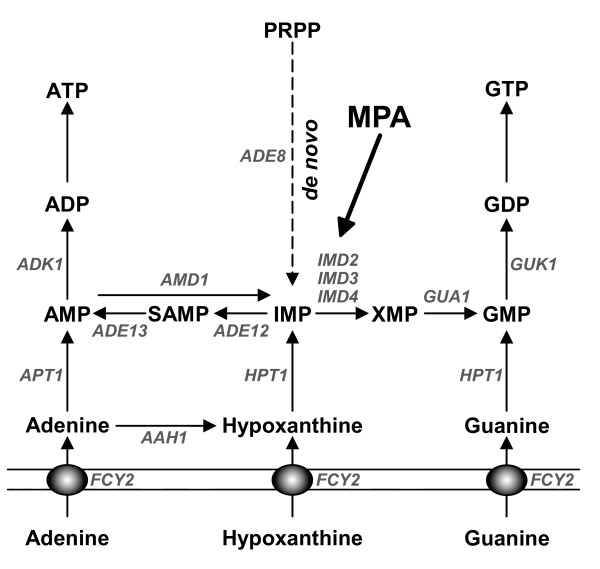
**Schematic representation of the purine nucleotide synthesis pathway in yeast**. The solid thin lines represent the plasma membrane. ADP: adenosine-5'-diphosphate; AMP: adenosine-5'-monophosphate; ATP: adenosine-5'-triphosphate; GDP: guanosine-5'-diphosphate; GMP: guanosine-5'-monophosphate; GTP: guanosine-5'-triphosphate; IMP: inosine-5'-monophosphate; PRPP: 5-phosphoribosyl-1-pyrophosphate; SAMP: S-adenosine-5'-monophosphate; XMP: xanthosine-5'-monophosphate. Genes are shown in grey italic font and encode the following enzymatic activities: *AAH1*: adenine deaminase; *ADE8*: 5'-phosphoribosylglycinamide formyltransferase; *ADE12*: adenylosuccinate synthetase; *ADE13*: adenylosuccinate lyase; *ADK1*: AMP kinase; *AMD1*: AMP deaminase; *APT1*: adenine phosphoribosyltransferase; *FCY2*: purine cytosine permease; *GUA1*: GMP synthetase; *GUK1*: GMP kinase; *HPT1*: hypoxanthine-guanine phosphoribosyltransferase; *IMD2*, *IMD3*, and *IMD4*: IMP dehydrogenases (*IMD1 *is not indicated here because it is not expressed and is thought to be a pseudogene). Mycophenolic acid (MPA) inhibits IMP dehydrogenases.

To generate guanylic nucleotides, the requirement for IMPDH activity can be bypassed by the addition of guanine into the growth medium (Fig. 1). To show that the effects of MPA on cell cycle progression specifically result from a decrease of intracellular guanylic nucleotide pools, we treated cells with MPA in guanine-supplemented growth medium. In these conditions, only 3% of the cells displayed two unseparated daughters (Fig. 3) and no growth defect was detected (data not shown and [18]).

As 100 μg/mL MPA causes a significant decrease of the translation efficiency [18], we considered the possibility that "bibudded" cells result from impaired translation. To this end, we examined cells treated for 4 hours with the general translational inhibitor cycloheximide at a concentration that abolished translation [20]. In contrast to MPA-treated cells, cycloheximide-treated cells did not exhibit more than one bud (0.8% ± 0.3% of the budding cells had two buds, N>200). Therefore, by decreasing intracellular guanylic nucleotide pools, MPA treatment caused a slowing down of the yeast cell cycle progression, the telophase being mostly affected. Consequently, "bibudded" cells accumulate and this, independently of MPA effects on translation efficiency.

### Mutations affecting the guanylic nucleotide biosynthesis cause the emergence of cells with two unseparated daughters

**Figure 4:**
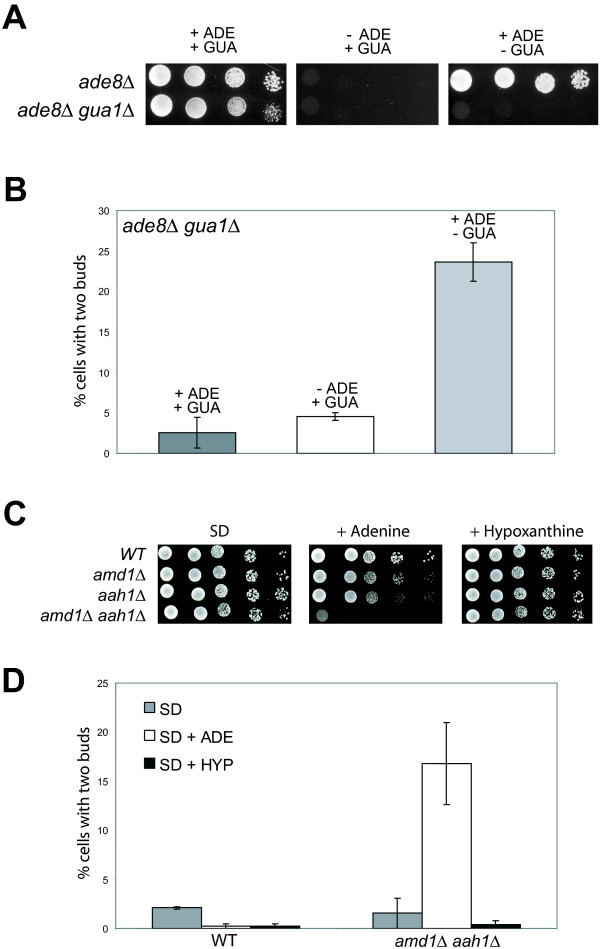
**Effects of guanylic nucleotide starvation using mutants of the purine nucleotide biosynthesis pathway**. A. Growth of an *ade8*Δ *gua1*Δ double mutant and an isogenic *ade8*Δ single mutant on SD medium containing the indicated purines. The SD + ADE + GUA medium contains 25% Adenine / 75% Guanine ratio (total purine concentration of 0.3 mM). **B**. The *ade8*Δ *gua1*Δ strains was grown in SD medium containing 25% Adenine / 75% Guanine ratio to OD_600 nm _= 0.2; then shifted into the indicated medium. After 4 hours of incubation at 30°C, the percentage of cells with two daughters among budding cells was counted. More than 200 cells were counted for each condition. **C**. Growth of the WT strain, isogenic single *amd1*Δ or *aah1*Δ mutant strains and *amd1*Δ *aah1*Δ double mutant strain on SD medium, SD medium supplemented with adenine or SD medium supplemented with hypoxanthine. **D**. WT strain (left) and *amd1*Δ *aah1*Δ double mutant strain (right) were grown in SD medium to OD_600 nm _= 0.2 and then shifted into the indicated medium. After 4 hours of incubation at 30°C, the percentage of cells with two daughters among budding cells was counted. More than 200 cells were counted for each condition.

Although addition of guanine to the growth medium fully reversed the effects of MPA, pharmacological studies have the caveat of possible secondary targets. Thus, we developed mutant yeast strains in which the guanylic nucleotide pools can be modulated by a simple change of the growth conditions. Because of genetic redundancy in IMPDH encoding genes, no single *imd *mutant is auxotroph for guanine, we therefore chose to use a mutant in the *GUA1 *gene which encodes GMP synthetase, the enzyme converting XMP into GMP (Fig. 1). A *gua1*Δ mutant is unable to synthesize guanylic nucleotides in the absence of guanine in the growth medium. To compare the effects of guanylic nucleotide starvation with the depletion of another purine, we combined the *gua1*Δ deletion with the *ade8*Δ deletion that leads to adenine auxotrophy. The double mutant *ade8*Δ *gua1*Δ is thus auxotroph for both guanine and adenine (Fig. 4A). When both guanine and adenine were provided in the growth medium, the double mutant *ade8*Δ *gua1*Δ grew like the isogenic *ade8*Δ control strain and, the *ade8*Δ *gua1*Δ population contained less than 3% of "bibudded" cells (Fig. 4A and 4B). After a 4-hour shift to a growth medium lacking guanine, 20% of the cells displayed two unseparated daughter cells. By contrast, shifting the cells to growth medium lacking adenine did not induce the emergence of "bibudded" cells (Fig. 4B). Therefore, "bibudded" cells appearance is specific to a guanylic nucleotide starvation.

In order to diminish the intracellular guanylic nucleotide pools by another route, we constructed an *aah1*Δ *amd1*Δ double mutant. The *AAH1 *gene encodes adenine amino-hydrolase, an enzyme converting adenine into hypoxanthine. The *AMD1 *gene encodes the AMP deaminase enzyme, which converts AMP into IMP (Fig. 1). The *de novo *synthesis of IMP from PRPP is inhibited by the presence of adenine in the growth medium [21]. Consequently, in the presence of adenine, the *aah1*Δ *amd1*Δ double mutant has theoretically no path to synthesize GMP, neither from the *de novo *pathway nor from adenine. Accordingly, without extracellular guanine (or hypoxanthine), the guanylic nucleotide pools of the *aah1*Δ *amd1*Δ double mutant cannot be replenished and its growth is strongly affected (Fig. 4C). The *aah1*Δ *amd1*Δ double mutant was grown in SD medium without purine to OD_600 nm _= 0.2 and then shifted into a medium containing adenine as a sole source of purine. After 4 hours, the percentage of cells with two unseparated buds was counted. As shown in figure 4D, "bibudded" cells only arose when the *aah1*Δ *amd1*Δ double mutant cells were grown in a medium containing adenine. Thus, modifying the guanylic nucleotide pools through mutations in the purine nucleotide biosynthesis pathway provoked the same effect that MPA treatment on the yeast cell cycle: drastically impaired cell separation without cell cycle arrest, resulting in cells with two unseparated daughters.

### Guanylic nucleotide starvation does not affect the formation of cellular structures required for completion of cytokinesis

The decrease of intracellular guanylic nucleotide pools caused the appearance of cells with two unseparated buds. We further characterized these abnormal cells to identify the cellular process(es) critically impaired by this starvation and thus likely responsible for this particular phenotype.

**Figure 5:**
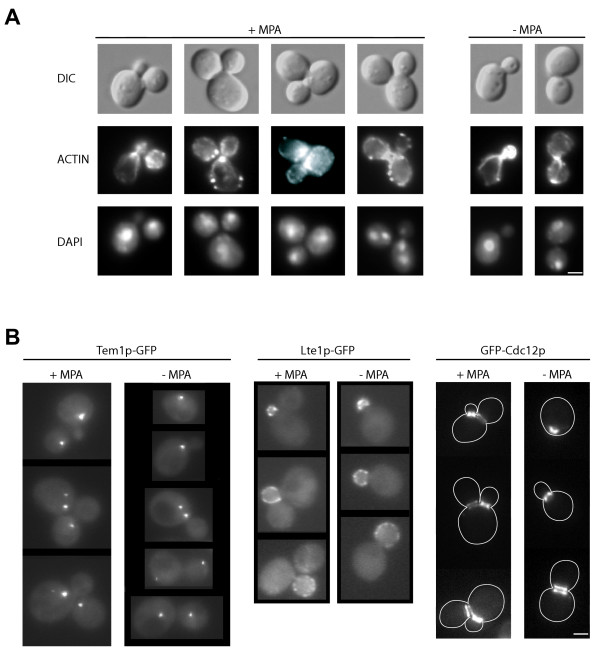
**Localization of several cellular structures or fusion proteins in cells with two daughters**. **A**. Cells were untreated (right panel) or treated (left panel) with 100 μg/mL MPA for 4 hours. DIC (top lane), actin Alexa-phalloidin staining (middle lane) and DAPI staining (bottom lane) are shown. **B**. Localization of endogenous Tem1p-GFP (left panel), endogenous Lte1p-GFP (middle panel) and GFP-Cdc12p expressed from a centromeric plasmid under the control of its own promoter (right panel) in cells untreated (right of each panel) or treated for 4 hours with 100 μg/mL MPA (left of each panel). Only cells displaying two buds are shown in the case of MPA treatment. Bar: 2 μm.

When synchronized cells were treated with MPA, the timing of emergence and the size of the second bud compared to the first one (see Fig. 2E) strongly suggested that MPA treated cells started a new cell cycle before the separation of the first daughter cell. Cells with two daughters follow a normal budding pattern (Fig. 2E and 5A), and are morphologically very different from the multibudded cells observed for polarity mutants impaired in bud emergence. Thus, the decrease of intracellular guanylic nucleotide pools did not likely affect cell polarity establishment. Further, most of the cells with two buds presented wild type, polarized actin patches and cables (Fig. 5A, middle lane), confirming previous studies showing that MPA treatment does not drastically affect the actin cytoskeleton [18]. In addition, nuclei of the "bibudded" cells are properly positioned (Fig. 5A, bottom lane) although abnormal mitosis occurs in less than 5% of the cells (Fig. 5A, + MPA right panel). Thus, polarization establishment and nuclear segregation were not drastically affected in cells with two unseparated daughters.

Microscopic observation of synchronized MPA-treated cells revealed that although anaphase on-set was delayed, its duration was almost similar to the anaphase in control cells (Fig. 2B). Therefore, MPA treatment did not critically affect the mitotic exit network (MEN). Further, MPA treatment did not disrupt proper localization of Tem1p, a RAS GTPase governing the MEN, neither the localization of its GTP exchange factor (GEF) Lte1p (Fig. 5B, left and middle panels). Taken together, these results suggest that MPA treatment does not significantly perturb anaphase progression, and consequently does not activate the anaphase checkpoint.

Because MPA severely affected progression through telophase, we examined the formation of cellular structures essential for the completion of cytokinesis. Figure 5A shows that both the first and the second daughter cell can build an actin cytokinetic ring. Further, more than 95% of the MPA treated cells (N>200) maintain proper localization of the septin Cdc12p (Fig. 5B, right panel), suggesting strongly that MPA-induced guanylic nucleotide pools depletion does not affect the septin ring formation and stability. Moreover, in "bibudded" cells, the septin ring can split to allow acto-myosin ring contraction for both the first and the second daughter cell (Fig. 5B, right panel). Therefore, guanylic nucleotide starved cells displayed all the structures required for cytokinesis completion, and the cellular processes leading to the mother-daughter separation defect must occur later in the cell cycle.

### Guanylic nucleotide starvation affects cell wall separation

**Figure 6:**
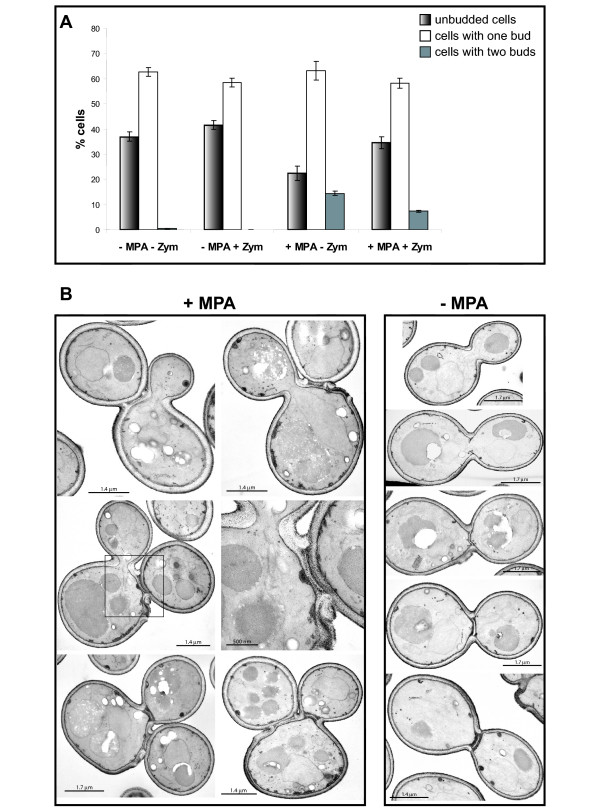
**Effects of MPA on the yeast cell wall**. **A**. Percentage of unbudded (shadow bars), single budded (white bars) and of cells with two buds (grey bars) after 4 hours treatment with 100 μg/mL MPA followed by a mild digestion of the cell wall with zymolyase. More than 200 cells were counted for each condition. **B**. Electron microscopy pictures of cells with two buds obtained after 4 hours treatment with 100 μg/mL MPA. Untreated control cells are shown on the right panel each steps of the septum formation are illustrated (from top to bottom).

As cells with two unseparated daughters properly form both actin and septin rings, we examined whether the cytoplasm was still continuous between the first bud and its mother. After a 4 hour MPA treatment, we digested the cell wall with zymolyase and counted the number of remaining "bibudded" cells. Figure 6A shows that mild treatment with zymolyase cause a decrease of the "bibudded" cell number suggesting strongly that in those cells, the cytoplasm is no more continuous between the mother cell and at least one daughter cell. Thus, guanylic nucleotide depletion apparently did not affect cytoplasm constriction but rather a later step in the daughter cell separation process. To confirm this result, we observed MPA-treated cells by electron microscopy after Thiéry coloration, which reveals polysaccharides and therefore the cell wall. As shown in figure 6B, the cytoplasm between the mother and the first daughter cell is no longer continuous. Thus, guanylic nucleotide starvation did not affect cytoplasm closure but a later step in the daughter cell separation process. Further, the secondary septum of the first daughter cell appears normal by electron microscopy, although some small *lacunae *were occasionally observed (see inset of Fig. 6B). Therefore, we speculate that reduction of intracellular guanylic nucleotide pools by MPA treatment particularly impinges on the separation of the daughter cell by affecting the cell wall digestion.

### A large proportion of cells entering quiescence exhibit two daughters

**Figure 7:**
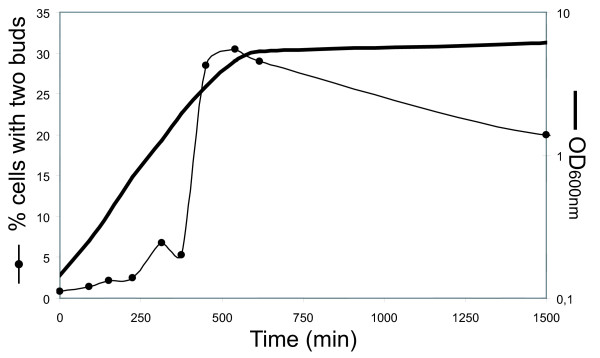
**Number of cells with two daughters as a function of the age of the culture**. WT cells were grown in SC medium and the percentage of cells with two daughters among budding cells as a function of time was counted (black circles). For each time point, more than 300 cells were counted. OD_600 nm _is indicated as a black bold line.

In the course of our study, we noticed that a small but reproducible amount of untreated wild type cells displayed two unseparated buds (see for example Fig. 3), a morphology identical to MPA-treated "bibudded" cells. We then examined the frequency of "bibudded" cells during the growth of a wild type yeast population. To our surprise, whereas less than 2% of the cells with two unseparated buds could be observed during exponential phase, when yeast approached the diauxic shift, more than 30% of the budding population presented two apparent daughter cells (Fig. 7). This observation suggested a slowing of daughter cell separation upon the last divisions before stationary phase. Like previously, we verified the authenticity of the "bibudded" phenotype by mixing two populations of cells, each expressing one different GFP variant. When this mixed population reached the diauxic shift, we counted the number of cells with two apparent daughters and examined them by fluorescence microscopy. Similar to our results for MPA-treated cells, only 0.9% ± 0.7% of the apparent "bibudded" cells were bicolor (more than 200 "bibudded" cells were counted for each experiment). Thus, we concluded that those cells were indeed cells with two daughters. This was further supported by observation of those cells by electron microscopy (not shown). We obtained similar results with both the BY4742 and the FL100 genetic background (data not shown) demonstrating that the appearance of cells with two daughters is not specific to the BY4742 background. In conclusion, when a yeast culture approaches stationary phase, a significant proportion of cells behaves like cells in which the intracellular guanylic nucleotide pools have been depleted and give rise to cells with two unseparated daughters.

## Discussion

Guanylic nucleotides are not only "building blocks" for nucleic acids but are also crucial for the regulation of many cellular processes such as G-proteins based signaling pathways. Therefore, cells must maintain their concentrations at a critical level. However, the molecular relationships between intracellular guanylic nucleotide levels and cell proliferation crucial events remain poorly understood.

Here, we have demonstrated that MPA treatment does not cause a firm cell cycle arrest in yeast. Treated cells continue to proliferate, although at a reduced rate (this study and [18]). Further, we have shown that conditional mutants unable to synthesize guanylic nucleotides do not arrest in a particular stage of the cell cycle. Therefore, our results establish that there is no guanylic nucleotide checkpoint is *S. cerevisiae*. By contrast, in mammalian cells, it was previously shown that MPA treatment cause an arrest of cellular proliferation but no guanylic nucleotide specific checkpoint has clearly been identified. Several reports have described that MPA treatment affects mammalian cells ability to commit into division by blocking the transition from G0 to the S phase of the cell cycle [13,17,22]. However, if MPA is added when cells have already entered the S phase, the cell cycle arrest occurs in G2/M [22]. Besides, the MPA-induced arrest is not fully reversed by the replenishment of guanylic nucleotide pools [13,17,22]. Here, we have shown that even if MPA treatment slows all stages of the yeast cell cycle progression, the most affected step is the mother-daughter cell wall separation, giving rise to "bibudded" cells. This result supports our previous observation that MPA treatment leads to the appearance of many cells with 3N DNA content [18]. Importantly, MPA treatment has the same effects on both non-synchronized and synchronized cells treated with the drug upon release from G1 (Fig. 2) or upon release from G2/M after nocodazole synchronization (I. S., B. D.-F. unpublished results). Therefore, unlike in mammals, in yeast, MPA treatment causes the same effects whatever the cell cycle stage of the cells at the time of drug addition. Further, our analysis of mutants demonstrates that the mother-daughter separation defect results solely from guanylic nucleotide pools depletion and is independent of potential MPA secondary targets.

In yeast, the fact that a guanylic nucleotides starvation causes a mother-daughter separation defect was unexpected. Indeed, one could have intuitively supposed that this depletion would rather provoke a drastic defect during DNA replication upon S phase or disturb cell cycle steps for which GTPase driven molecular processes are essential. In fact, upon MPA treatment, cells can still build a daughter cell and cell polarity was found even less affected in this study than in our earlier work [18]. Daughter cell appears to grow normally, mitosis proceeds unperturbed, and mother-daughter closure is properly achieved. Thus, guanylic nucleotides starvation does not critically affect the functions of key GTP binding proteins, such as Cdc42p, Tem1p, tubulin and septins. In this study we observed that in the "bibudded" cells, placement of the second daughter cell properly follows the axial budding pattern of haploid cells, suggesting that the bud-site selection machinery is properly located. In contrast, prolonged MPA treatment (48 hours) leads to a random budding pattern [18]. Thus, long-term guanylic nucleotides starvation may have more drastic effects. Most importantly, our experiments show that complete mother-daughter separation is not required for the mother cell to pass through START and to generate a second daughter cell (Fig. 2E). Therefore, our data confirm that no additional checkpoint blocks the cell cycle progression when anaphase is properly achieved.

What molecular targets trigger the mother-daughter separation defect upon guanylic nucleotide starvation? Observation of "bibudded" cells by electron microscopy revealed no obvious defect in the overall septum architecture of abnormally unseparated daughter cell. Nevertheless, MPA treatment increases cells sensitivity to SDS [19], zymolyase or sonication (I. S., B. D.-F. unpublished results), strongly suggesting cell wall defects. In fact, previous works have illustrated links between guanylic nucleotides metabolism and cell wall integrity, particularly through the synthesis of mannoproteins, essential components of the fungal cell wall. GDP-mannose is the common substrate for mannosyltransferases, enzymes catalyzing the addition of mannose residues on mannoproteins core oligosaccharides. In budding yeast, the GDP-mannose pyrophosphorylase Psa1p is an essential enzyme that synthesizes GDP-mannose, using GTP as a substrate. It was previously shown that MPA treatment affects Psa1p expression [18] and that Psa1p depletion leads to cell separation failure [23]. Further, Shimma et al have demonstrated that the major defect of a *guk1 *conditional mutant strain, that is impaired for GDP biosynthesis (Fig. 1), was a decrease in GDP-mannose level (to about 25% of the wild type levels) that leads to mannose outer chain elongation defects [24]. Accordingly, we have observed cell separation defect in *guk1 *cells (I. S., B. D.-F., unpublished results). In addition, yeast lacking mannosyltransferase encoding genes, such as *OCH1*, *MNN10 *or *ANP1 *display both hypersensitivity to MPA [19] and a cell separation defect similar to the one observed for guanylic nucleotide starved cells [25-27]. Therefore, one major consequence of guanylic nucleotide starvation could be a significant decrease in the GDP-mannose pool that in turn leads to a mother-daughter separation defect.

The most intriguing aspect of the regulation of intracellular guanylic nucleotide pools is the correlation between the IMPDH activity and cellular proliferation in mammalians models. Interestingly, the transcription of the *IMD2 *gene is actively shut off *via *regulatory sequences when yeast cells enter stationary phase upon nutrients limitation [2]. Transcriptome analyses have demonstrated that *AAH1*, *HPT1 *and *GUA1 *are among the most promptly down regulated genes when nutrients become limiting. Thus, it appears that an entire process is devoted to rapidly decrease the intracellular guanylic nucleotide pools when cells enter stationary phase. Here, we have demonstrated that the typical "bibudded" phenotype obtained during guanylic nucleotide starvation also occurs in untreated wild type cells achieving their last divisions upon nutrients limitation. Thus, an identical morphology is observed for both guanylic nucleotide starvation and entry into quiescence.

Finally, the GTP/GDP ratio is very sensitive to growth conditions, rapidly decreasing during the diauxic shift and drastically dropping upon nutrients starvation. Further, this ratio may regulate RAS GTPases activity by influencing its guanylic nucleotide loading equilibrium [11]. RAS and TOR pathways are key regulators that coordinate yeast proliferation with nutrients availability. Recent work has suggested that the TOR protein acts as ATP sensor in mammals [28]. Thus it is appealing to speculate that in parallel to the TOR pathway, intracellular guanylic nucleotides levels are part of a signal that regulate cell proliferation via the modulation of RAS GTPases activity.

## Conclusion

Using either mycophenolic acid, a molecule that specifically inhibits the first committed step in *de novo *GMP biosynthesis or mutations in the guanylic nucleotide biosynthesis pathway, we have demonstrated that intracellular guanylic nucleotides limitation causes a mother-daughter cell wall separation defect in budding yeast. This defect leads to the emergence of cells with two unseparated daughters. These "bibudded" cells are also found in a population of cells entering quiescence upon nutrient limitation. These observations further suggest that guanylic nucleotide intracellular pools might contribute to a signal that regulates cell proliferation, particularly upon nutrients limitation and entry into stationary phase.

## Methods

### Strains, media and reagents

Yeast strains used were purchased from Euroscarf (Frankfurt, Germany) and are derivatives of BY4741 or BY4742 [29]. SD and SD casa media were described previously [2] and were supplemented when required with tryptophan (0.2 mM), uracil (1.8 mM), guanine (0.3 mM), adenine (0.3 mM), hypoxanthine (0.3 mM). Cycloheximide was purchased from Sigma and used at 50 μg/mL. Mycophenolic acid (MPA) was purchased from Amresco, (Ohio, USA) and was used at 100 μg/mL.

### Plasmids

pB1594 integrates three tandem copies of GFP at the 3' end of the *LTE1 *coding region (Lte1p-3xGFP) and is a kind gift of D. Pellman [30]. pB1598 integrates three tandem copies of GFP at the 3' end of the *TEM1 *coding region (Tem1p-3xGFP) and is a kind gift of D. Pellman. pYB407 (GFP-Cdc12p – *LEU2 *– CEN) is a kind gift of Y. Barral [31]. pVTYS65T (*URA3*, 2μ) allows the expression of the GFP variant containing the S65T mutant (fluorescence maxima: Ex: 488 nm, Em: 511 nm) and has been previously described [32]. pVTYBFP2 allows the expression of a GFP variant containing mutations F64L, S65T, Y66H et Y145F which coding sequence has been optimized for expression in yeast (fluorescence maxima: Ex 380 nm, Em 440 nm see [33]). Details of the mutagenesis and constructions are available upon request.

### Cell Biology techniques and Fluorescence Microscopy

Cells were synchronized by addition of alpha-factor at the final concentration of 10 μg/mL directly into the growth medium. After 3 hours of incubation at 30°C with agitation, more than 99% of the cells were unbudded. Cells were washed twice and released into fresh SD medium containing or not MPA. Propidium iodide (Sigma) staining was performed as described in [34] except that cells were fixed with ethanol 70%. Alexa-568-Phalloidin (Molecular Probes, Eugene, OR) was used to stain filamentous actin as described previously [35]. Zymolyase 20T (ICN Biomedicals, Costa Mesa, CA) was used at 0.2 mg/mL on formaldehyde fixed cells resuspended in PBS. Images were acquired with a Marianas system and analyzed with the Slidebook software (Intelligent Imaging Innovations, Inc. Denver, CO) except for GFP-Cdc12p expressing cells that were imaged with a previously described imaging system [32].

### Electron Microscopy

Cells were grown in SD medium to OD_600 nm _= 0.2 at 30°C. MPA was added to the final concentration of 100 μg/mL and cells were grown for four more hours. Cells were then fixed with glutaraldehyde and osmic acid, and then stained by the method of Thiéry as previously described [36].

## Authors' contributions

JS carried out the electron microscopy. IS did the other experimental work. BDF and IS conceived and coordinated the studies and drafted the manuscript. All authors read and approved the final manuscript.
